# Impaired behavioral flexibility and coping strategy shifts in 5xFAD mice in the novel Destruction-Building Test

**DOI:** 10.3389/fnbeh.2026.1877086

**Published:** 2026-08-26

**Authors:** Eugenia Ahremenko, Alexander Andreev, Kirill Chaprov, Eduard Korkotian

**Affiliations:** 1 Faculty of Chemistry, Perm State University, Perm, Russia; 2 Institute of Physics and Mathematics, Perm State University, Perm, Russia; 3 Institute of Physiologically Active Compounds, Federal Research Center of Problems of Chemical Physics and Medicinal Chemistry, Russian Academy of Sciences, Chernogolovka, Russia; 4 Department of Brain Sciences, The Weizmann Institute of Science, Rehovot, Israel

**Keywords:** 5XFAD mice, Activities of Daily Living (ADLs), behavioral assays, Destruction-Building Test (DBT), spontaneous locomotor activity (SLA), manipulative performance, sex differences

## Abstract

**Introduction:**

The assessment of early ethological markers of neurodegeneration in Alzheimer’s disease (AD) remains a pivotal task in preclinical research. This study evaluates the diagnostic potential of the “Destruction-Building Test” (DBT), a novel assay based on the innate manipulative behaviors of rodents–specifically material shredding and complex nest-building–as an early indicator of neurodegenerative changes.

**Methods:**

Investigations were conducted on hemizygous 5xFAD transgenic mice and wild-type (WT) controls of both sexes at 15, 28, and 42 weeks of age. The protocol included a 24-h habituation phase with continuous actigraphy to assess spontaneous locomotor activity (SLA), followed by a 72-h phase of manipulative activity. Generalized Estimating Equations (GEE) were employed for statistical modeling.

**Results:**

We observed a systemic reduction in manipulative activity in 5xFAD mice, which was most pronounced in females starting from 15 weeks of age. GEE modeling confirmed that genotype and sex were the primary determinants of shredding deficits (*p* < 10^–9^). Furthermore, we identified a robust negative correlation between SLA during the initial adaptation phase and subsequent manipulative productivity in the DBT (*p* < 0.001); specifically, higher exploratory locomotion predicted less efficient shredding and nest-building. Transgenic animals demonstrated a pathological shift toward a proactive coping style characterized by unproductive hyperlocomotion and diminished manipulation, signaling impaired behavioral flexibility and cognitive rigidity.

**Discussion:**

These findings suggest that the DBT is highly sensitive to early neurodegenerative manifestations before the onset of gross motor deficits.Its methodological simplicity and robust diagnostic value position the DBT as an effective tool for the longitudinal screening of cognitive status in AD mouse models.

## Introduction

The use of animal models remains a fundamental tool in developing therapeutic strategies for neurodegenerative disorders, particularly Alzheimer’s disease (AD) (Arakawa and Iguchi, 2018). Transgenic mouse lines expressing mutant proteins associated with familial AD allow for the reconstruction of key pathological features and preclinical screening (Yokoyama et al., 2022; Lopes et al., 2025). The 5xFAD line is recognized as one of the most aggressive AD models, harboring five specific mutations in the APP and PSEN1 genes (Yokoyama et al., 2022). These mutations initiate the overproduction of Aβ_42_ starting at 1.5–2 months of age, leading to the formation of extensive amyloid plaques, microgliosis, neuroinflammation, synaptic loss, and advancing cognitive and motor dysfunctions (Forner et al., 2021; Pádua et al., 2024). Crucially, by 4 months of age (15 weeks), these mice manifest well-documented synaptic density reduction and early memory decline in the absence of gross motor impairments, making this specific age milestone a critical window for identifying early functional biomarkers.

To assess the behavioral phenotype in AD models, traditional assays such as Novel Object Recognition (NOR), the Y-maze, and the Morris Water Maze (MWM) are commonly employed. However, results across different laboratories often demonstrate low reproducibility, with conflicting reports of significant memory deficits versus preserved performance in comparable age groups of 5xFAD mice (Wei et al., 2016; Hüttenrauch et al., 2017; O’Leary and Brown, 2022; Choi et al., 2023; Faisal et al., 2023; Lansdell et al., 2023; Sánchez et al., 2023; O’Leary and Brown, 2024; Zhu and Liu, 2025). Such variability is driven by a lack of protocol standardization, high stress in unfamiliar environments, investigator interference, and environmental variables (Schellinck et al., 2010; Gulinello et al., 2019; Genzel, 2021; The International Brain Laboratory, Aguillon-Rodriguez et al., 2021; Saré et al., 2021; Lang et al., 2023). The use of insensitive methodologies risks false-negative results and unjustified expenditure of preclinical resources (Sukoff Rizzo and Silverman, 2016; Papanastasiou et al., 2025). Furthermore, most conventional methodologies rely on binary assessments (Galati et al., 2025) and ignore rodent’s endogenous motives in favor of anthropomorphic perceptions of cognitive function (Arakawa and Iguchi, 2018; Kondrakiewicz et al., 2019; Genzel, 2021; Puścian and Knapska, 2022; Lang et al., 2023; Török and Bárdos, 2026).

Consequently, there is an increasing need for low-stress assays that allow animals to express instinctive behaviors within their home-cage environment (Dennis et al., 2021; Rosenberg et al., 2021; d’Isa and Gerlai, 2023; Lang et al., 2023). Nest construction and substrate manipulation are evolutionarily ancient, highly conserved behaviors vital for thermoregulation, protection, and welfare (Estep et al., 1975; Deeming, 2023; Tagawa et al., 2025). Nest-building deficits in 5xFAD mice emerge as early as 4–5 months and correlate with neuropathology (O’Leary and Brown, 2024; Keszycki et al., 2023). Importantly, these complex species-typical home-cage behaviors are not merely automated motor outputs; they heavily rely on cortical-hippocampal pathways, spatial orientation, executive planning, and sustained motivation (Deacon, 2006; Jirkof, 2014). Assessing material shredding and nest construction thus provides an objective proxy for executive integrity, motivation, and functional status (Barnett and Hocking, 1981; Keszycki et al., 2023).

In this study, we present the Destruction-Building Test (DBT) - a comprehensive methodology integrating the assessment of nest-building behavior with a quantitative analysis of material-shredding activity within a framework of continuous long-term multichannel actigraphy. Operating as an uninterrupted 96-h (4-day) protocol where the animal remains undisturbed within the testing unit, the DBT incorporates an initial 24-h habituation phase (Day 0) to record baseline spontaneous locomotor activity (SLA) before introducing a destructible substrate for a 72-h manipulative phase (Days 1–3). The aim of this research is to validate the DBT in the 5xFAD line across longitudinal lifespan stages (15, 28, and 42 weeks of age) and determine its sensitivity in identifying age-related differences between transgenic and control animals for preclinical drug screening.

## Materials and methods

### Animals and housing conditions

The study utilized transgenic 5xFAD mice [B6-Tg(APPSwFlLon, PSEN1*M146L*L286V)6799Vas/J] and their non-transgenic siblings (wild-type, WT) on a C57Bl/6J background. Originally mice line JAX #006554 obtained from the Jackson Laboratory was on a C57BL/6 × SJL hybrid background and backcrossed to C57BL/6J more than at 20 generations. Animals were maintained under SPF conditions at the Center for Collective Use of the Institute of Physiologically Active Compounds (IPAC RAS), Russian Academy of Sciences (contract #FFSG-2024–0020). The 5xFAD line is characterized by the expression of human APP and PSEN1 genes harboring five mutations: Swedish (K670N/M671L), Florida (I716V), and London (V717I) in the APP gene, as well as M146L and L286V in the PSEN1 gene.

Mice were housed in groups of 3–5 per cage (type 1284L, Tecniplast, Italy) under standard conditions: a 12-h light/dark cycle, temperature of 19 °C–24 °C, humidity of 25%–65%, and *ad libitum* access to food and water.

### Genotyping

The genotype of all animals was confirmed both before and after the experiment using PCR. DNA was extracted from ear biopsies. To identify the transgenic *APP* cassette located on chromosome 3, we used a common forward primer 5′-ACCCCCATGTCAGAGTTCCT-3′, a mutant reverse primer 5′-CGGGCCTCTTCGCTATTAC-3′, and a wild-type reverse primer 5′-TATACAACCTTGGGGGATGG-3′, resulting in fragments of 129 bp for the mutant tg and 216 bp for the WT.

The PSEN1 insertion was identified using internal control primers oIMR1644 5′-AATAGAGAACGGCAGGAGCA-3′ and oIMR1645 5′- GCCATGAGGGCACTAATCAT-3′, alongside oIMR7338 5′-CTAGGCCACAGAATTGAAAGATCT-3′ and oIMR7339 5′-GTAGGTGGAAATTCTAGCATCATCC-3′, yielding a 608 bp transgene fragment and a 324 bp internal positive control.

Given the high prevalence of the Pde6b mutation (retinal degeneration 1, rd1) in hybrid lines and its potential impact on behavioral results, all animals were additionally screened for the rd1 allele. Carriers and homozygotes for the rd1 mutation were excluded from the study. The primers used were 5′-CTACAGCCCCTCTCCAAGGTTTATAG-3′ (common forward), 5′-ACCTGCATGTGAACCCAGTATTCTATC-3′ (mutant reverse), and 5′-AAGCTAGCTGCAGTAACGCCATTT-3′ (wild-type reverse), producing fragments of 560 bp (mutant) and 240 bp (WT). PCR was performed using a standard 35-cycle amplification program: 95 °C (15 s), 65 °C (15 s), and 72 °C (15 s) on a Bioer GeneExplorer Thermal Cycler (GE-96G, China).

### Ethical statement

The number of animals used was minimized in accordance with the 3Rs principles. All animal procedures were performed in accordance with the European Communities Council Directive on the care and use of laboratory animals (2010/63/EU) and Russian regulations on animal protection. The study was approved by the Local Bioethics Committee of the Center for Preclinical Testing IPAC RAS (Protocol No. 101, 16 December 2024) and was conducted in line with ARRIVE guidelines (accessed 1 March 2026).

### Experimental design and group allocation

Following weaning at postnatal day 30 (P30), mice were housed in their home cages in groups of 3–5 individuals. Groups were formed based on sex, with wild-type (WT) and 5xFAD (Hem) animals co-housed within the same cages to minimize the influence of environmental factors on intergroup differences. Behavioral testing was conducted using a strict within-subject longitudinal design, wherein the exact same cohorts of animals (Wild-Type and 5xFAD transgenics) were sequentially tracked and evaluated at three successive lifetime milestones corresponding to the progressive stages of amyloid pathology: 15, 28, and 42 weeks of age. This sequential within-subject architecture eliminated inter-individual baseline variability. Detailed information regarding the active number of animals per group and per age point across the longitudinal timeline is provided in Table 1.

**TABLE 1:** Distribution of animals across experimental groups (*N* = 59).

Sex	Genotype	Group	15 weeks (*n*)	28 weeks (*n*)	42 weeks (*n*)
Females	Wild type	WT	14	10	5
	Transgenic	5xFAD (Hem)	15	10	5
Males	Wild type	WT	16	12	7
	Transgenic	5xFAD (Hem)	14	10	6
Total			59	42	23

*N*, total number of unique individual animals enrolled at the baseline of the study; *n*, number of active subjects evaluated at a specific lifetime milestone. The sequential reduction in sample size (*n*) within the cohorts across successive time points was driven by a predefined, staggered tissue-harvesting protocol. Specifically, a designated subgroup of animals (*n* = 10 per sex/genotype) was pre-allocated for sacrifice immediately following behavioral tracking at the 15 and 28-week milestones to harvest brain tissues for parallel histological, morphological, and immunohistochemical (IHC) analyses. Natural mortality characteristic of advanced neurodegenerative pathology in the 5xFAD line contributed to the sample size reduction.

### Behavioral testing: Destruction-Building Test

#### Equipment and video analysis

The study was conducted using the MultiNeuro-SLA software and hardware complex (OpenScience, Russia), which consists of 32 individual recording chambers (IRCs). The T3-PC type chambers (ZoonLab, Germany) were equipped with video cameras and infrared (IR) illumination. Video stream processing and differential tracking, centered on the point between the pinnae, were performed using the AsioOtus Lab software suite based on the DeepLabCut neural network engine. The total trajectory length was utilized as a quantitative measure of spontaneous locomotor activity (SLA).

#### DBT procedure

The automated DBT protocol was run as a continuous, uninterrupted 96-h (4-day) assay conducted under standard housing conditions, with constant twilight illumination (1–2 lux, white light). Continuous 24-h behavioral tracking on Day 0 and video recording throughout the full 96-h period were maintained seamlessly, thereby completely eliminating human handling stress and investigator interference during the testing cycle. The test consisted of two distinct, sequential yet integrated stages:

During Day 0 (Integrated Home-Cage Habituation and Baseline SLA Phase), animals were placed individually in the IRCs with *ad libitum* access to food and water. The baseline SLA in the novel environment was recorded during this initial 24-h period. Crucially, no nest-building or destructible materials were present during this initial phase, allowing spontaneous locomotor activity to stabilize into a baseline circadian rhythm and eliminating potential behavioral competition between exploratory locomotion and manipulative activity.

From Day 1 to Day 3 (Manipulative Activity Phase), a destructible object was introduced into the cages: a peat pot serving as a shredding substrate. The substrate consisted of dry pressed peat cardboard in the shape of a truncated pyramid, with square top and bottom bases measuring 40 and 70 mm, respectively, and a lateral face height of 75 mm. Additionally, a non-woven polyester fiber wipe (25.5 × 20.5 cm) was provided. The animals could utilize shredded pot fragments as structural material, integrating them into the spatial matrix of the wipe. Upon completion of the 4-day cycle, the nests were removed for weighing and morphometric analysis.

Before each experimental run, standard aerosol disinfection was performed using Nocospray (OxyPharm, France). Examples of mouse interactions with these objects and the resulting structures are provided in the Supplementary Figures 1–3, 5. For all 4 days of the experiment (96 h), the animals were continuously housed in the MultiNeuroSLA hardware and software system. The experimental cycle began and ended in the evening (around 8 p.m.).

#### Data collection and documentation

Peat pots were weighed and photographed under IR illumination with a 160° field of view every 24 h (at the end of each Day). At the end of Day 3 (the 96-h mark), the animals were removed from the setup, and their body weight was recorded. The nests formed by the mice (see Supplementary Figure 4) were collected, weighed, and photographed in three projections (initial state and unfolded from both sides) under standardized lighting. To assess metabolic status and exclude potential motor defects of the masticatory apparatus, daily food consumption was monitored by weighing 2–3 provided pellets and the subsequent remains.

#### Recorded parameters

Based on the data collected, the following parameters were analyzed: peat pot weight dynamics as an indicator of manipulative performance intensity, the shredded surface area of the pots as a morphometric measure of gnawing, and the final nest mass. Additionally, the analysis included spontaneous locomotor activity, the area of pot fragments integrated into the nest structure, and the average daily food consumption.

#### Image analysis

Image processing and standardization were performed using the Krita graphic editor. Quantitative surface area analysis was conducted within the ImageJ (version 1.54p) utilizing the Labkit segmentation plugin.

#### Statistical analysis

Statistical processing was performed using the R programming language (R Core Team, 2023). The selection of statistical methods was rigorously guided by the non-parametric distribution of most ethological variables, variance heteroscedasticity, and the repeated-measures structure of our within-subject longitudinal design, which featured shifting sample sizes (*n*) across milestones.

To account for the longitudinal data structure and handle intra-individual correlations without assuming strict normality or balanced groups, Generalized Estimating Equations (GEE) with an exchangeable working correlation structure were utilized as the primary mathematical modeling framework (Zeger et al., 1988). The GEE analytical pipeline was formally audited and validated under the expert supervision of a biostatistician (Prof. Marina A. Barulina, DSc in Physics and Mathematics, Director of the Institute of Physics and Mathematics, Perm State University). GEE was preferred over traditional repeated-measures ANOVA because it provides robust population-averaged estimates that are highly resilient to missing data points caused by sequential animal sacrifices or natural mortality.

Preliminary distributional testing was conducted using the Shapiro–Wilk test. For variables violating the normality assumption, non-parametric analyses were applied. For the evaluation of dynamic parameters, including cumulative changes in peat pot weight and surface area, as well as nest characteristics, a robust one-way or two-way ANOVA was applied. Pairwise intergroup comparisons *(post hoc* analysis) and the assessment of single parameters, such as locomotor activity and final nest mass, were conducted using the Wilcoxon test; *p*-values were adjusted for multiple comparisons using the Holm method.

#### Correlation analysis

The relationship between behavioral metrics was evaluated using the Spearman rank correlation coefficient.

#### Mathematical modeling

To identify the factors determining the intensity of manipulative performance intensity, Generalized Estimating Equations (GEE) with an exchangeable working correlation structure were utilized (Zeger et al., 1988). This method accounted for the longitudinal data structure and provided robust standard error estimates for repeated measures. Prior to model construction, predictors were screened for multicollinearity by calculating the Variance Inflation Factor (VIF) (Kim, 2019).

#### Software tools

Primary processing of the actigraphy data was performed using the AsioOtus Lab software suite.

## Results

### Dynamics of manipulative performance in the DBT

**FIGURE 1:**
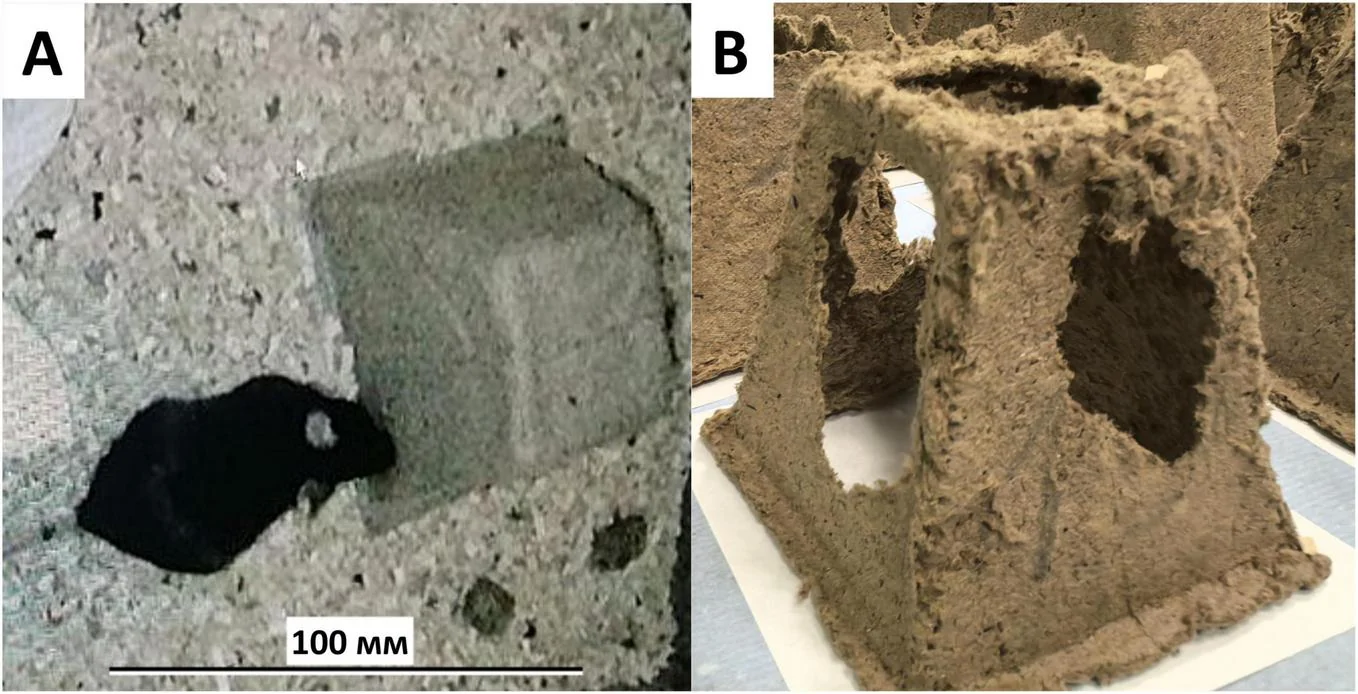
A mouse during the Destruction-Building Test (DBT) and the typical appearance of a peat pot after test completion. **(A)** Photograph of a mouse in the process of shredding the pot. **(B)** The image presents the characteristic destruction of the material resulting from the animal’s manipulative activity during the 72-h experimental period.

**FIGURE 2:**
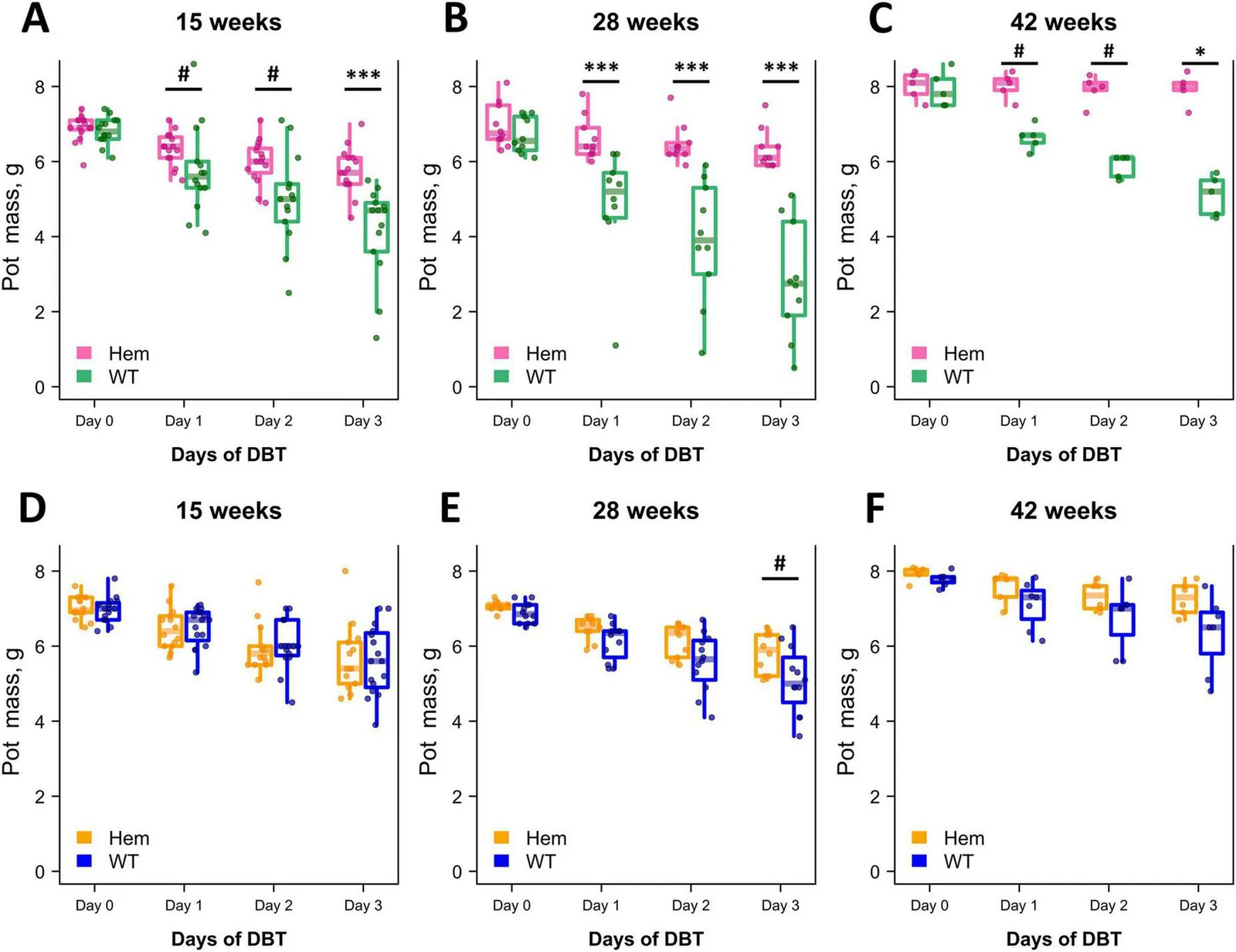
Dynamics of peat pot weight changes during the 72-h Manipulative Activity Phase of Destruction-Building Test (DBT). **(A–C)** Present data for females, and **(D–F)** for males. Group color coding: transgenic 5xFAD (Hem) mice - pink (females) and orange (males); wild-type (WT) control group - green (females) and blue (males). The ordinate represents pot weight (g), and the abscissa represents time (Base - initial mass, Day 1–3 - experimental days). **(A,D)** correspond to 15 weeks of age, **(B,E)** to 28 weeks, and **(C,F)** to 42 weeks. Colored dots represent individual data points for each subject; due to identical values or perfect alignment, some data points may overlap with each other or coincide with the median line, appearing as a single marker. Horizontal lines within the plots represent medians. Significance levels: #*p* < 0.05; *p < 0.01; ***p < 0.0001.

The intensity of shredding activity was evaluated by measuring the changes in the mass of the peat pot over the 3-day (from Day 1 to Day 3, 72 h) experimental period. A typical appearance following the animal’s interaction with the pot is shown in Figure 1. Generalized data for pot weight dynamics across all age points are presented in Figure 2.

Females: Analysis using a robust mixed two-way ANOVA revealed a significant effect of genotype on pot mass at all investigated time points: 15 weeks (*p* = 0.005), 28 weeks (*p* = 0.004), and 42 weeks (*p* = 0.0052). At the age of 15 weeks, significant differences between WT and 5xFAD groups were recorded at the end of Day 1 of the DBT test (*p* > 0.05) and increased until the conclusion of the experiment (Figure 2A and Table 2). In the 28- and 42-week testing sessions, a statistically significant decrease in pot mass in transgenic females compared to controls was observed as early as 24 h after the start Manipulative Activity Phase (*p* < 0.0001 for 28 weeks; *p* < 0.05 for 42 weeks). By 42 weeks, manipulative performance in 5xFAD mice was almost completely lost: the pot mass remained close to the baseline, while control animals continued to actively shred the object (Figure 2C).

**TABLE 2:** Results of pairwise comparisons of peat pot mass (Wilcoxon test).

Age	Sex	Day 1			Day 2			Day 3		
		5xFAD (Hem), means ± SEM	WT, means ± SEM	5xFAD (Hem) vs. WT (*p*)	5xFAD (Hem), means ± SEM	WT, means ± SEM	5xFAD (Hem) vs. WT (*p*)	5xFAD (Hem), means ± SEM	WT, means ± SEM	5xFAD (Hem) vs. WT (*p*)
15 weeks	Females	6.33 ± 0.11	5.76 ± 0.31	**0.023**	5.99 ± 0.15	4.99 ± 0.33	**0.011**	5.75 ± 0.16	4.16 ± 0.33	**0.000**
	Males	6.46 ± 0.15	6.51 ± 0.13	0.59	5.95 ± 0.18	6.05 ± 0.17	0.21	5.66 ± 0.24	5.61 ± 0.23	0.97
28 weeks	Females	6.61 ± 0.18	4.88 ± 0.47	**0.00055**	6.47 ± 0.16	3.89 ± 0.51	**0.0002**	6.31 ± 0.17	2.84 ± 0.48	**0.0002**
	Males	6.47 ± 0.1	6.13 ± 0.14	0.062	6.16 ± 0.14	5.58 ± 0.22	0.064	5.83 ± 0.17	5.08 ± 0.25	**0.034**
42 weeks	Females	8.02 ± 0.15	6.64 ± 0.15	**0.012**	7.92 ± 0.17	5.88 ± 0.14	**0.011**	7.94 ± 0.18	5.1 ± 0.24	**0.008**
	Males	7.58 ± 0.16	7.1 ± 0.24	0.17	7.33 ± 0.15	6.74 ± 0.31	0.31	7.27 ± 0.18	6.33 ± 0.38	0.062

Statistically significant differences (*p* < 0.05) are indicated in bold. The mean values of the masses of peat pots and standard errors of the means (SEM) are presented in grams (g).

Males: In contrast to females, robust ANOVA revealed no statistically significant effect of genotype on pot weight dynamics in any of the age testing sessions (*p* > 0.05). Although a general trend toward decreased pot mass was observed in all groups (Figures 2D–F), significant differences between WT and 5xFAD were recorded only at 28 weeks of age on Day 3 of the test (pairwise Wilcoxon test, *p* < 0.05). At 42 weeks, a visual divergence of medians was observed (with control group boxplots shifting downward); however, due to high inter-individual variability and sample size, these differences did not reach the threshold of statistical significance.

The overall level of manipulative activity in males was substantially weaker than in females. For instance, by the 28th week, many control females had almost completely destroyed the pot by Day 3, whereas in males, the mass of the object rarely fell below 4 g (50% of the initial weight).

### Analysis of material destruction area

**FIGURE 3:**
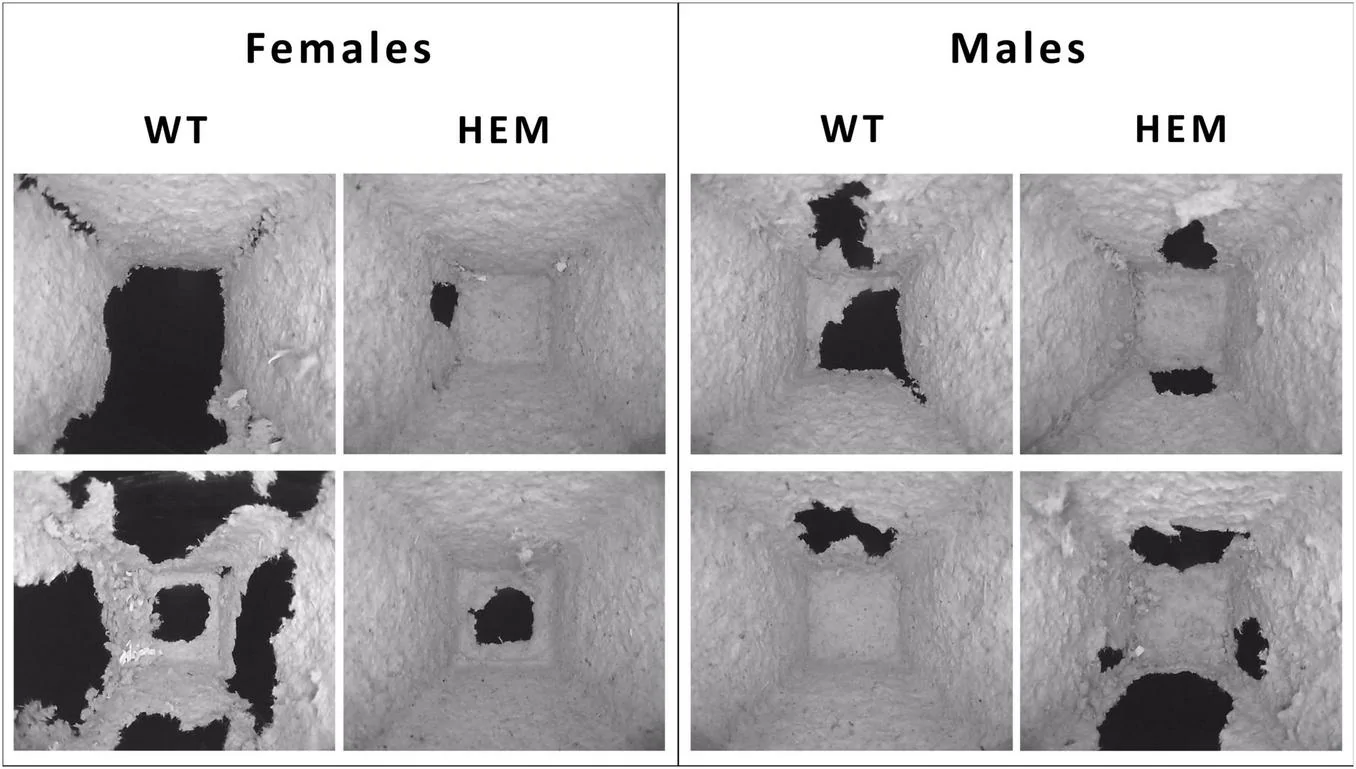
Character of peat pot destruction (internal view) in mice at 28 weeks of age. Typical examples of the objects are presented on the Day 2 of the experiment. From left to right: a wild-type (WT) female demonstrating pronounced shredding of the walls and bottom; a transgenic (Hem) female with minimal signs of manipulation; a wild-type (WT) male and a transgenic (Hem) male exhibiting moderate and similar manipulative activity.

**FIGURE 4:**
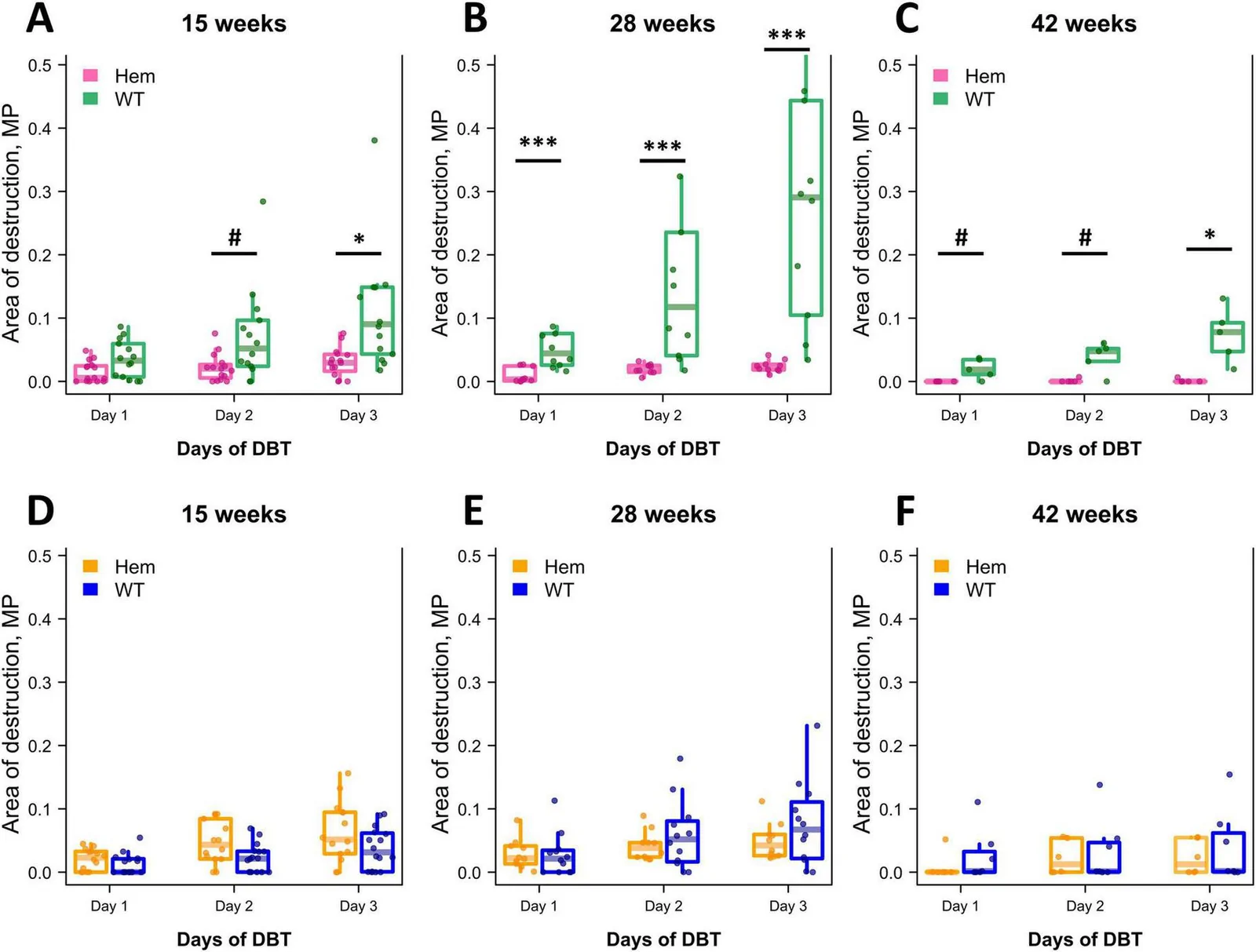
Dynamics of changes in the destruction area of peat pots during the 72-h Manipulative Activity Phase of Destruction-Building Test (DBT). **(A–C)** Present data for females, and **(D–F)** for males. Group color coding: transgenic 5xFAD (Hem) mice - pink (females) and orange (males); wild-type (WT) control group - green (females) and blue (males). The ordinate represents the area of the destroyed part (Mpix), and the abscissa represents time (Day 1–3 - experimental days). **(A,D)** correspond to 15 weeks of age, **(B,E)** to 28 weeks, and **(C)** to 42 weeks. Colored dots represent individual data points for each subject; due to identical values or perfect alignment, some data points may overlap with each other or coincide with the median line, appearing as a single marker. Horizontal lines within the plots represent medians. Significance levels: #*p* < 0.05; *p < 0.01; ***p < 0.0001.

The degree of object destruction is clearly illustrated by typical examples in Figure 3 (for both female and male groups of both genotypes). The results of the morphometric analysis of the destroyed pot area are presented in Figure 4 and Table 3.

**TABLE 3:** Results of pairwise comparisons of peat pot destruction areas (Wilcoxon test).

Age	Sex	Day 1			Day 2			Day 3		
		5xFAD (Hem), means ± SEM	WT, means ± SEM	5xFAD (Hem) vs. WT (*p*)	5xFAD (Hem), means ± SEM	WT, means ± SEM	5xFAD (Hem) vs. WT (*p*)	5xFAD (Hem), means ± SEM	WT, means ± SEM	5xFAD (Hem) vs. WT (*p*)
15 weeks	Females	0.02 ± 0	0.03 ± 0.01	0.07	0.02 ± 0.01	0.07 ± 0.02	**0.026**	0.03 ± 0.01	0.15 ± 0.05	**0.001**
	Males	0.02 ± 0	0.01 ± 0	0.12	0.05 ± 0.01	0.02 ± 0.01	0.06	0.06 ± 0.01	0.04 ± 0.01	0.16
28 weeks	Females	0.01 ± 0	0.11 ± 0.06	**0.00073**	0.02 ± 0	0.18 ± 0.06	**0.00021**	0.02 ± 0	0.29 ± 0.06	**0.00004**
	Males	0.03 ± 0.01	0.03 ± 0.01	0.54	0.04 ± 0.01	0.06 ± 0.02	0.77	0.05 ± 0.01	0.08 ± 0.02	0.5
42 weeks	Females	0 ± 0	0.02 ± 0.01	**0.032**	0 ± 0	0.04 ± 0.01	**0.032**	0 ± 0	0.07 ± 0.02	**0.008**
	Males	0.01 ± 0.01	0.03 ± 0.02	0.18	0.02 ± 0.01	0.03 ± 0.02	0.84	0.02 ± 0.01	0.04 ± 0.02	0.63

Statistically significant differences (*p* < 0.05) are indicated in bold. Area values were obtained using automated image analysis of the interior of the objects. The mean values of destruction areas and standard errors of the means (SEM) are presented in megapixels (MP).

Females: Robust mixed two-way ANOVA confirmed a significant effect of genotype on the destruction area in females at 15 weeks (*p* = 0.005), 28 weeks (*p* = 0.004), and 42 weeks (*p* = 0.0052). For the female group, significant differences between genotypes were recorded as early as 15 weeks of age, becoming evident by the end of Day 2 of the test (*p* < 0.05), with a subsequent increase in significance toward the end of the exposure (Table 3). In older age points, the deficit in manipulative performance in transgenic individuals was already apparent by the end of Day 1 of the experiment (*p* < 0.0001 for 28 weeks; *p* < 0.05 for 42 weeks) (Figures 4A–C). A pronounced age-related dynamic was observed: the destruction area in transgenic females progressively decreased, reaching a complete loss of manipulative activity by the 42nd week (differences between the 28- and 42-week time points for transgenic female: F = 35.9, *p* < 0.001).

Males: No statistically significant differences were found for this parameter in males, except for a borderline value at 15 weeks of age (*p* = 0.056). In contrast to females, the destruction areas in the WT and 5xFAD male groups were nearly identical (Figures 4D–F). The only deviation was recorded on Day 2 at 15 weeks of age, where transgenic individuals showed a slightly larger destruction area than the control; however, this effect was not sustained.

A comparative analysis of the sexes showed that the total area of destruction in males was substantially lower than in females. These data are consistent with the results of the gravimetric analysis, confirming a higher intensity of interaction with the object in females. It is worth noting that differences in pot mass in the transgenic female group were more pronounced than differences in the area of the openings. It is likely that in the early stages of pathology, the animals retain the ability to gnaw the material but do not always chew through it completely, which hinders the visual detection of defects.

In control females, an increase in the destruction area was observed during repeated testing at a more mature age, which may indicate the accumulation of experience or an increase in ethological motivation. No such effect was observed in males. A methodological limitation of the current setup configuration must be considered: the resolution of the cameras and the viewing angle allow for reliable recording of destruction only in the bottom section and the adjacent wall areas up to three-quarters of the total height, leaving the rim region outside the scope of accurate analysis. However, gravimetric analysis (weighing) fully compensates for this visual gap.

### Characteristics of constructed nests in the DBT

**FIGURE 5:**
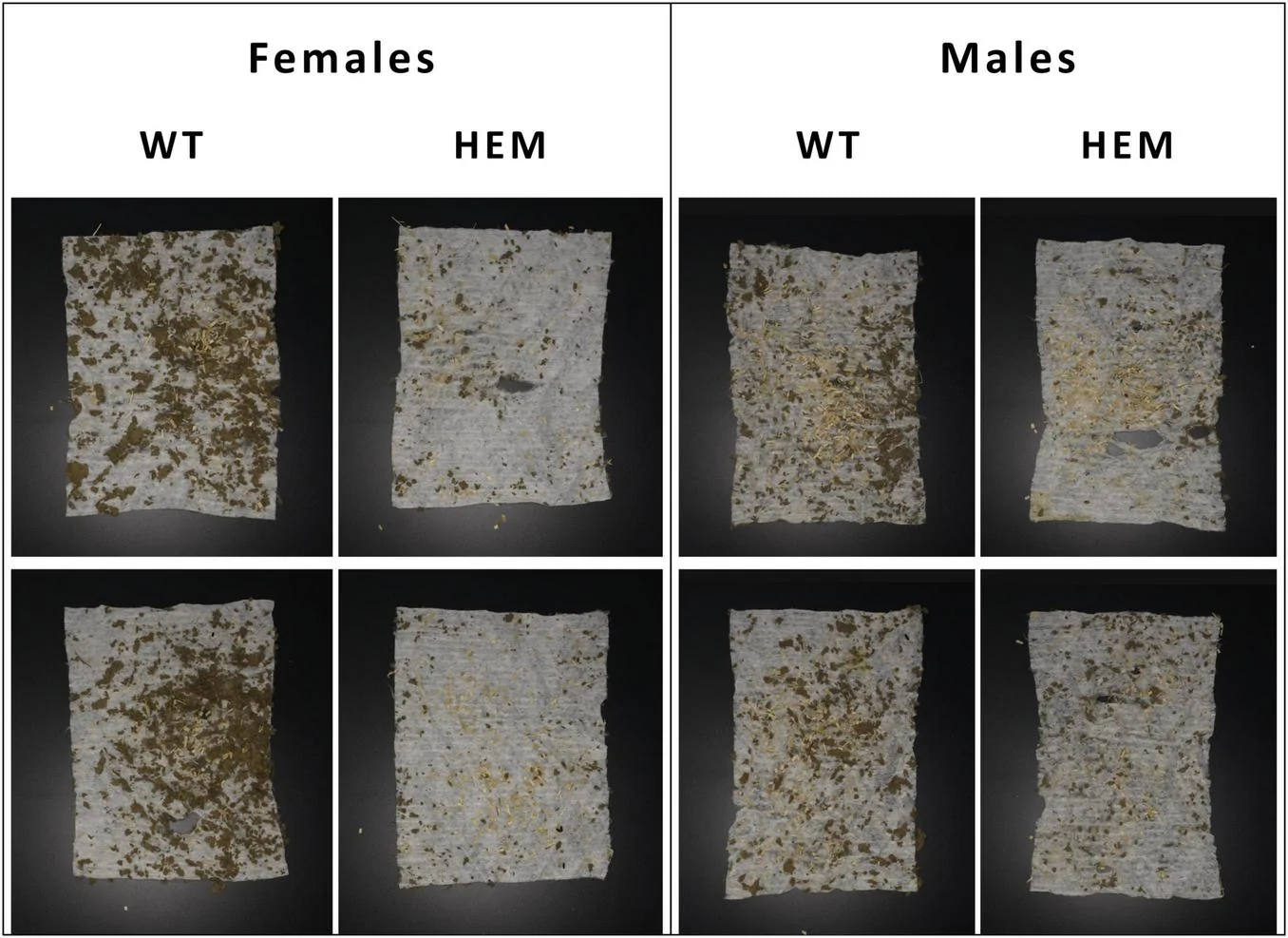
Examples of composite nest architecture in mice at 28 weeks of age. The images display the integration of the non-woven base (napkins) and the peat substrate (pot fragments). From left to right: a nest of a wild-type (WT) female with a high degree of material fragmentation; a nest of a transgenic (Hem) female characterized by minimal pot destruction and low-level processing of the napkin; examples of nests from a wild-type (WT) male and a transgenic (Hem) male demonstrating an intermediate level of manipulative activity.

**FIGURE 6:**
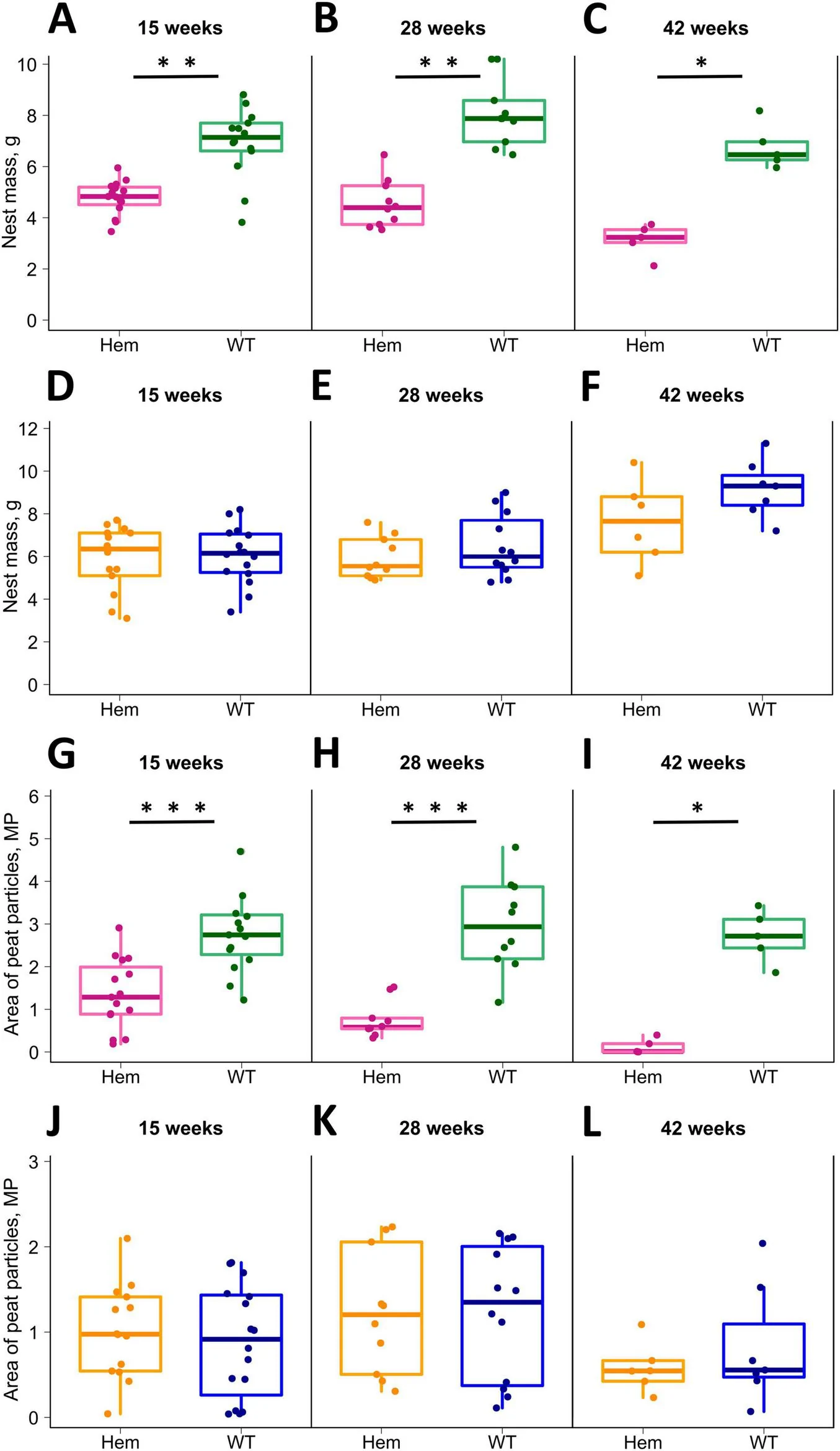
Comparative analysis of nest mass and the area of incorporated elements in WT and 5xFAD (Hem) mice. **(A–F)** Present data on the mass of constructed nests (g); **(G–L)** show the total area of pot fragments woven into the nest structure (Mpix). Age points: **(A,D,G,J)** - 15 weeks; **(B,E,H,K)** - 28 weeks; **(C,F,I,L)** - 42 weeks. Group color coding: Control (WT) - green (females) and blue (males); transgenic animals (Hem) - pink (females) and orange (males). Graphical notation: Individual values for each subject are shown as dots of the corresponding group color. Statistics: *P*-values were obtained using the Wilcoxon *post-hoc* test. Significance levels: *p < 0.01; **p < 0.001; ***p < 0.0001.

The quantitative assessment of the nests was performed based on two key parameters: the final mass and the total area of the peat pot fragments incorporated into the napkin. Examples of composite nests (peat crumbs and non-woven matrix) are presented in Figure 5. Statistical data for the mass and area of the pot elements are shown in Figure 6.

Females: Pairwise comparison (Wilcoxon test) revealed significant differences between WT and 5xFAD mice across all age points. Nest mass in control females was significantly higher at 15 weeks (*p* = 0.0002), 28 weeks (*p* = 0.00021), and 42 weeks (*p* = 0.008). A similar pattern was observed for the total area of incorporated elements: 15 weeks (*p* = 0.0001), 28 weeks (*p* = 0.00004), and 42 weeks (*p* = 0.008). The nests of control animals were characterized by their massiveness and structural complexity due to the active inclusion of fragments from the destroyed pot (Figures 6A–C, G–I). In contrast, transgenic females barely used pot material in their construction, which correlates with the previously described deficit in manipulative performance.

Males: Nests of males from both genotypes were virtually identical across both parameters. At 42 weeks of age, a weak trend toward increased nest mass was observed in control individuals compared to transgenic ones (Figure 6F); however, this effect did not reach statistical significance. Regarding the area of incorporated elements, no differences were found between WT and 5xFAD males (Figures 6J–L).

Comparative analysis showed that the nest mass of control males was comparable to that of control females, and at 42 weeks, it even exceeded it in some individuals. This indicates that basic nest-building behavior (interaction with the napkin) is preserved in males. However, building strategies differ between the sexes: males are less inclined to combine the non-woven base with the peat substrate. The identified deficit in transgenic females indicates an impairment in the ability to form complex, multi-component nests even in the early stages of pathology. This may serve as an indicator of a general cognitive deficit or a decrease in ethological motivation. Our data support literature reports indicating that in the 5xFAD line, the pathological phenotype in females appears earlier and is more pronounced (Manji et al., 2019; Bouter et al., 2021; Neuharth et al., 2025). The lack of pronounced differences in males may be due to a slower progression of the disease or qualitative differences in the manifestation of the pathology, necessitating further research in later age ranges.

The age-related dynamics for each DBT parameter for every individual group of mice are presented in Supplementary Figures 6–9.

### Spontaneous locomotor activity and its influence on shredding intensity: experimental data and mathematical modeling

#### Assessment of age-related dynamics of SLA

Assessment of spontaneous locomotor activity (SLA) was conducted during Day 0 of testing (the first 24 h) in individual actigraphic chambers. The interaction of the mice with the nesting/shredding materials (the DBT) took place from Day 1 to Day 3 (the subsequent 72 h), which allowed for the exclusion of direct competition between locomotion and manipulative behavior for the animal’s time resources.

**FIGURE 7:**
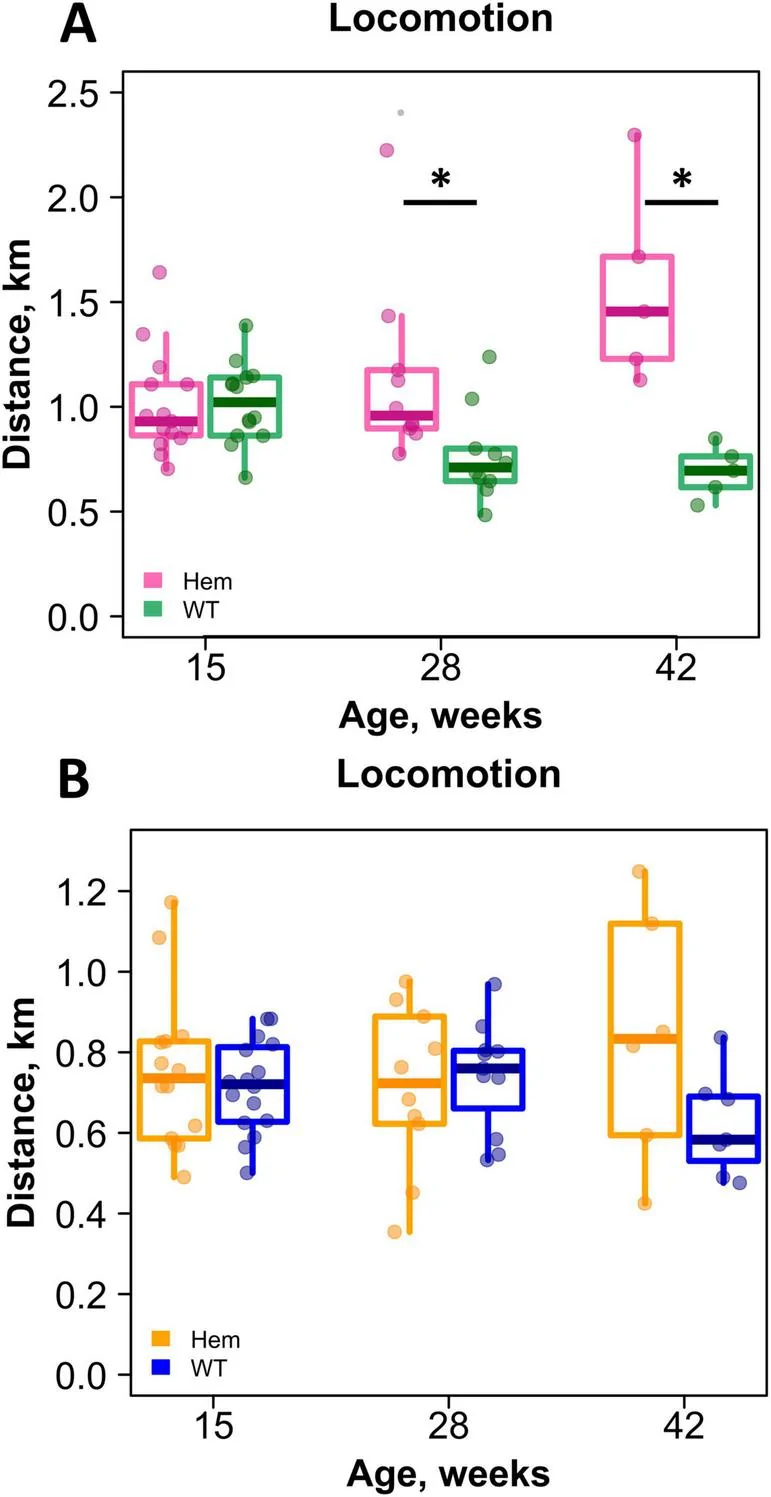
Dynamics of daily spontaneous locomotor activity (SLA). The panels present the total distance traveled over 24 h of habituation phase. *p < 0.01. **(A)** (Females): comparison of 5xFAD (pink outline, Hem) and wild-type (green outline, WT) groups. **(B)** (Males): comparison of 5xFAD (orange outline, Hem) and wild-type (blue outline, WT) groups. The ordinate (Y) indicates the distance traveled (km), and the abscissa (X) indicates the age of the animals in weeks. Colored dots represent individual data points for each subject. Error bars represent the 95% confidence interval.

Analysis of the age-related dynamics of SLA revealed sex differences in the accumulation of behavioral deviations. Transgenic 5xFAD females exhibited pronounced hyperlocomotion with age compared to the wild-type control group, which normally shows decreased locomotor activity during aging (Figure 7A). In males, this effect was less pronounced (Figure 7B). Significant differences in SLA between female groups were recorded at 28 weeks (*p* = 0.007) and 42 weeks (*p* = 0.008).

Despite genotypic differences in SLA dynamics, all groups were characterized by a negative correlation between locomotion on Day 0 and shredding intensity on subsequent days (Days 1–3): WT females (*r* = −0.52, *p* = 0.005), 5xFAD females (*r* = −0.63, *p* = 0.0001), WT males (*r* = −0.12, *p* = 0.5; effect not significant), and 5xFAD males (*r* = −0.38, *p* = 0.038). This correlation analysis indicates that increased locomotion during the adaptation period predicts a decrease in manipulative activity. However, a multifactorial model was required for a more accurate assessment of the SLA contribution.

#### Mathematical modeling of the influence of factors on shredding

To identify relationships between SLA and shredding intensity, a Generalized Estimating Equations (GEE) model was applied (see Supplementary material, programming code), which is suitable for analyzing longitudinal data with repeated (intra-individual) measurements. GEE accounts for the internal correlation of repeated measurements through an exchangeable structure, providing robust population estimates. Fixed-effect predictors included SLA (continuous variable), genotype, sex, and time point (categorical). In the first stage, a saturated model with all interactions was constructed. In this model, genotype, sex, and SLA were significant, but age was not.

Genotype as a cluster of interactions was highly significant (Wald χ^2^ = 39.2, *p* ≈ 3.8 × 10^–10^), demonstrating a borderline direct effect (*p* = 0.0901) and strong mediated effects through sex (χ^2^ = 27.0, *p* ≈ 2.0 × 10^–7^) and SLA (χ^2^ = 4.7, *p* = 0.030). Sex as a cluster was significant (Wald χ^2^ = 10.6, *p* = 0.0011), with a reduction in shredding in males. SLA as a cluster showed a strong effect (Wald χ^2^ = 22.5, *p* ≈ 2.1 × 10^–6^), where an increase in distance was associated with a decrease in shredding. Age and its interactions were not significant (Wald χ^2^ = 0.7, *p* = 0.4008).

#### Multicollinearity diagnostics and model simplification

The complexity of the saturated model given the limited sample size led to multicollinearity (VIF > 5 for interactions), indicating instability of the coefficients. To optimize the model, it was simplified by retaining the main effects and the key “Genotype × Sex” interaction. In the simplified model, the VIF decreased to acceptable values: genotype = 2.66, sex = 4.93, SLA = 1.55, age = 1.12, and genotype × sex = 4.75.

#### Results of the simplified GEE model

In the final simplified GEE model, all main effects remained highly significant. For genotype, a pronounced decrease in shredding activity was recorded in 5xFAD mice (Wald χ^2^ = 39.2, *p* < 10^–9^). For sex, males demonstrated lower manipulative performance (Wald χ^2^ = 10.6, *p* = 0.0011). SLA also showed significance (Wald χ^2^ = 22.5, *p* ≈ 2.1 × 10^–6^), where an increase in distance on Day 0 predicts a decrease in shredding performance. Age remained statistically not significant (Wald χ^2^ = 0.7, *p* = 0.40). The interaction of Genotype × Sex was highly significant (Wald χ^2^ = 27.0, *p* ≈ 2.0 × 10^–7^), showing that the deficit in manipulative activity in pathology is significantly more pronounced in females.

#### Interpretation: behavioral flexibility and coping styles

The negative association between locomotion and shredding in the GEE model can be explained by the concept of individual coping styles. According to the classification (Clinton et al., 2022), SLA during the adaptation period allows for the division of animals into two types. The proactive style (Proactive/Bold) is characterized by high exploration speed and a tendency toward stereotypical behavior; such individuals quickly master the space (high SLA) but show low sensitivity to environmental details and weak manipulative activity. In our experiment, mice with minimal shredding correspond to this style. The reactive style (Reactive/Shy) involves cautious exploration associated with high cognitive flexibility; these animals take longer to adapt to the space but pay significantly more attention to objects, resulting in thorough shredding of the substrate and high-quality nest construction (high DBT indicators). This heightened proactivity in the 5xFAD group may stem from pathological disinhibition typical of advanced neurodegeneration.

The identified correlation suggests that a high level of locomotion prevents timely switching to manipulative activity. In transgenic animals (especially 5xFAD females), a pathological shift toward proactivity is observed, indicating a deficit in behavioral flexibility - the ability to inhibit baseline exploratory locomotion in favor of a task-specific manipulative behavior. Since impaired flexibility is an early sign of neurodegeneration (Uddin, 2021), the DBT allows for the effective identification of this marker through natural behavior with minimal stress.

### Analysis of food consumption during the testing period

**FIGURE 8:**
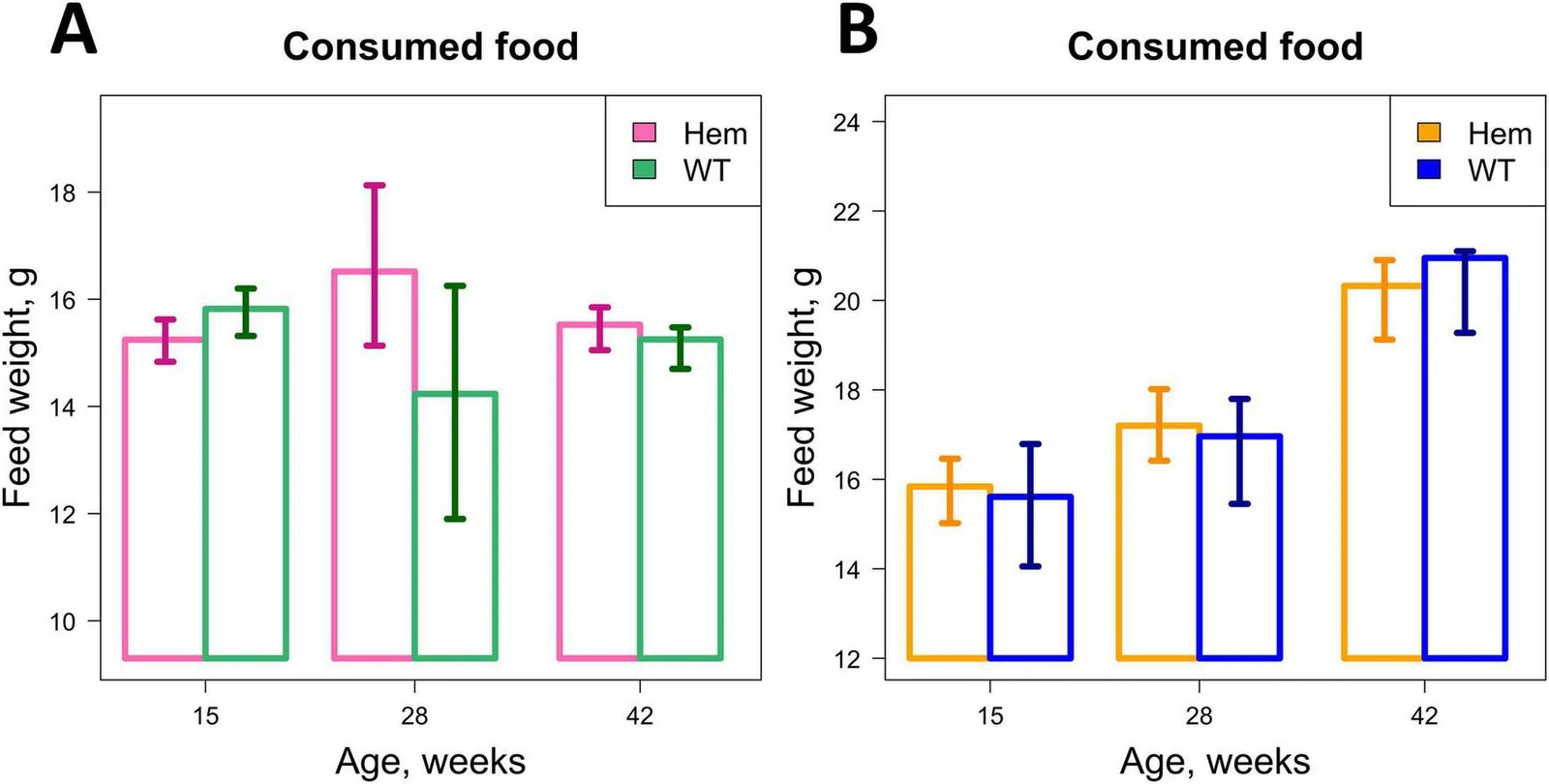
Average daily food consumption during the Destruction-Building Test (DBT) testing period. The panels present comparative data on the mass of food consumed over 24 h. **(A)** (Females): comparison of 5xFAD (pink outline, Hem) and wild-type (green outline, WT) groups. **(B)** (Males): comparison of 5xFAD (orange outline, Hem) and wild-type (blue outline, WT) groups. The abscissa indicates the age of the animals (weeks), and the ordinate indicates the mass of food consumed (g). Bars correspond to mean values, and error bars represent the 95% confidence interval.

To exclude the influence of potential chewing apparatus defects or a general decline in vitality on the DBT results, an assessment of the mass of food consumed during the entire period of individual housing was conducted. The results are presented in Figure 8.

Females: Statistical analysis (Wilcoxon test) revealed no significant differences in the volume of food consumed between the WT and 5xFAD groups. At the ages of 15 and 28 weeks, differences were observed at the level of a statistical trend (*p* = 0.054 and *p* = 0.059, respectively), but by the 42nd week, the indicators for both groups were virtually identical (*p* = 0.67) (Figure 8A).

Males: No statistically significant differences in the amount of food consumed were recorded in any of the age testing sessions (*p* > 0.05) (Figure 8B).

The preservation of the ability to effectively consume solid food (pellets) in transgenic animals indicates that the identified impairments in peat pot destruction are not related to dental pathology or jaw muscle dysfunction. The ability to gnaw as such remains intact.

Although a deficit in the DBT test could theoretically be associated with impaired fine motor skills of the forelimbs, according to literature data, pronounced motor impairments (coordination, reflexes, muscle strength) in the 5xFAD line manifest predominantly after 9 months of age (O’Leary et al., 2020; Gendron et al., 2021; Pádua et al., 2024; Valiantis et al., 2026). Thus, motor dysfunction is not the determining factor in the decline of nest-building abilities, at least at the earlier age points. This suggests that the phenotype observed in the DBT test is primarily due to the degradation of central mechanisms regulating complex goal-oriented behavior.

### Dynamics of animal body mass

Monitoring of animal body mass was conducted during the study from 15 to 42 weeks of age. Statistical analysis using a non-parametric two-way ANOVA (Rank two-way ANOVA) revealed a significant influence of genotype on body mass dynamics in both females (*p* = 2.3 × 10^–8^) and males (*p* = 2.6 × 10^–5^). To clarify the age periods in which intergroup differences manifest, a *post-hoc* analysis (Wilcoxon test) with Holm correction for multiple comparisons was performed.

Females: A significant decrease in body mass in transgenic individuals compared to controls was observed at all-time points: 15 weeks (*p* = 0.016), 28 weeks (*p* = 0.0015), and 42 weeks (*p* = 0.016).

Males: Significant differences were recorded at 15 weeks (*p* = 0.0014) and 28 weeks (*p* = 0.011). By the 42nd week, the gap between the groups narrowed and shifted into the category of a statistical trend (*p* = 0.094).

**FIGURE 9:**
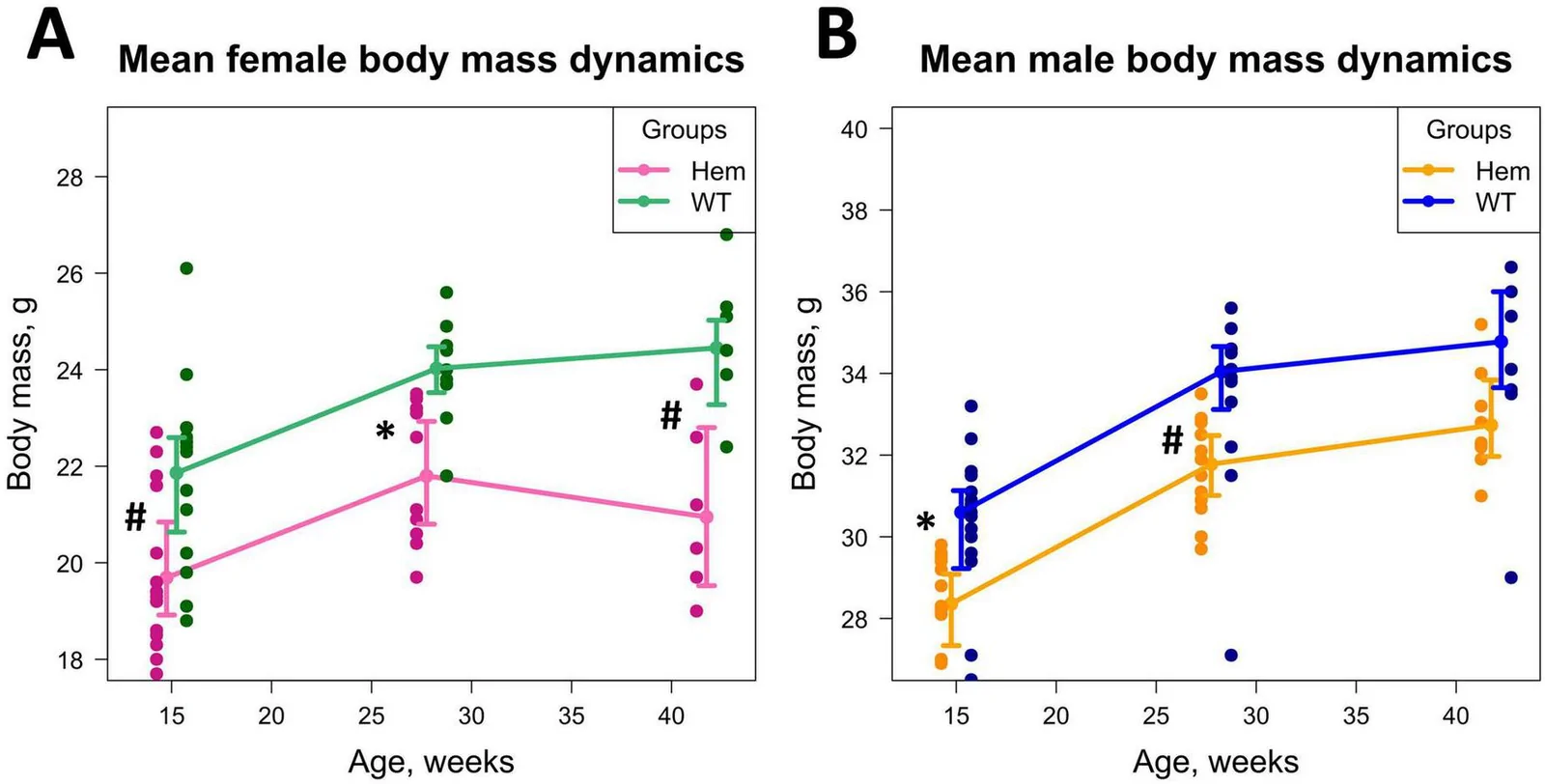
Dynamics of animal body mass. **(A)** Presents the body mass dynamics for females, and **(B)** for males. Color coding for wild-type (WT) groups: dark green (females) and dark blue (males). Color coding for transgenic groups (Hem): dark pink (females) and orange (males). The abscissa indicates age (weeks), and the ordinate indicates body mass (g). The graph lines connect the group mean values; individual points represent the data for each animal. Error bars correspond to the 95% confidence interval. Statistical significance: #*p* < 0.05; *p < 0.01.

In all studied groups and age points, transgenic animals (Hem) had lower body mass compared to control siblings (WT) (Figures 9A, B), consistent with previous reports (O’Leary et al., 2020; Oblak et al., 2021; Gendron et al., 2021; Forner et al., 2021). These data confirm the presence of systemic metabolic changes characteristic of the 5xFAD model, manifesting even at the early stages of the neurodegenerative process.

## Discussion

### Methodological advantages and ethological significance of the DBT

The Destruction-Building Test (DBT) developed here extends the classical Nest Building Test (NBT) methodology by expanding its diagnostic capabilities. While traditional NBT focuses primarily on the final nest quality, the DBT provides a quantitative assessment of its prerequisite behavioral components: manipulative performance (substrate shredding) and the capacity to integrate heterogeneous materials during active environmental transformation (Deacon, 2006; Angoa-Pérez et al., 2013; Kraeuter et al., 2019). Replacing subjective scoring scales with automated recording of physical parameters (material mass and fragment area) eliminates experimenter bias, which is often critical when evaluating subtle cognitive impairments (Neely et al., 2019; Dorninger et al., 2020).

Nest-building is an evolutionarily conserved instinct essential for thermoregulation, protection, and reproduction (Gaskill et al., 2013; Bárdos et al., 2022; Tagawa et al., 2025). The higher motivation for nest construction observed in control females is modulated by endogenous hormonal stimuli and sensory signals associated with maternal behavior (Tagawa et al., 2025), explaining the baseline sex differences in substrate interaction. Because nest-building is acutely sensitive to animal wellbeing - responding to stress, pain, or systemic inflammation (Jirkof, 2014; Tagawa et al., 2025) - its impairment serves as a robust indicator of cognitive decline in neurodegenerative models, including Down syndrome/AD and Parkinson’s disease (Heller et al., 2014; Robinson et al., 2024). Our findings in 5xFAD mice validate the DBT as a sensitive assay for early-stage neurodegeneration. To enhance diagnostic sensitivity in males, expanding the descriptor set for long-term manipulative tracking may be advisable.

Crucially, the multi-component nature of the deficits captured by the DBT should not be viewed as a confounding limitation, but rather as a key translational advantage. In clinical settings, prodromal and early stages of Alzheimer’s disease are fundamentally characterized by a progressive decline in Activities of Daily Living (ADLs) - complex, multi-step behavioral routines requiring the tight integration of executive planning, working memory, focused attention, and fine motor skills. While conventional rodent assays isolate narrow cognitive domains (e.g., spatial memory in the Morris Water Maze), they often fail to capture the cumulative functional collapse seen in patients. By leveraging the mouse’s innate drive to manipulate dense materials, the DBT serves as a direct murine analog to human ADLs, where performance failure reflects a generalized breakdown in executive drive and behavioral orchestration.

### Indicators of neurodegeneration in the DBT test

The present study demonstrates that the DBT provides sensitive behavioral descriptors to assess neurodegenerative progression in female 5xFAD mice. The assay showed high diagnostic sensitivity even at early pathology stages (15 weeks of age), when classical cognitive phenotypes often remain undetected in standard paradigms.

The primary marker of deficit was the collapse of complex goal-oriented activity: transgenic females exhibited reduced shredding intensity and failed to construct multi-component nests. While control animals actively transformed their environment -stretching the non-woven matrix and weaving peat elements into it -5xFAD mice virtually lost the capacity to integrate heterogeneous materials.

Furthermore, the DBT revealed a non-linear trajectory in intra-group phenotypic variance across disease progression in 5xFAD females. At 15 weeks, when amyloid pathology is in its nascent phase, the cohort remains relatively homogeneous, displaying low baseline variance. By 28 weeks, inter-individual variability peaks, marking a critical “phenotypic tipping point” characterized by an asynchronous onset of cognitive deficits. While some transgenic individuals maintain temporary functional compensation, others undergo a rapid collapse in manipulative performance, markedly inflating group variance. By 42 weeks, variability drops sharply due to a profound “floor effect,” as pathology reaches a plateau where surviving transgenic females converge at maximum functional impairment. This non-linear shift in variance demonstrates that the DBT captures not only the presence of deficits, but also the staging and population dynamics of neurodegeneration.

### Sensorimotor and ethological aspects of behavior

The behavioral deficit identified at 15 weeks cannot be attributed solely to gross motor dysfunction, as most studies in 5xFAD mice report impairments in locomotion and skeletal muscle strength primarily after 9 months of age (Pádua et al., 2024).

However, sensory impairments may play a contributing role. Existing data indicate an early decline in auditory and olfactory functions in the 5xFAD model (Na et al., 2023; Pádua et al., 2024; Su et al., 2026). Because animals were housed in a facility room housing breeding stock, control females may have heightened their nest-building behavior in response to ultrasonic vocalizations and olfactory cues from newborn pups (McRae et al., 2023; Tagawa et al., 2025). A blunted sensory responsiveness to such environmental stimuli in transgenic females might thus diminish their ethological drive for active environmental transformation.

### Cognitive dysfunction and shift in behavioral strategies

We hypothesize that nest-building impairments in 5xFAD mice stem primarily from deficits in executive functioning and cognitive flexibility. Executing this complex instinct requires sequential progression through planning, initiation, and sustained attention across an integrated behavioral chain (shredding → transportation → weaving) (Jirkof, 2014; Tagawa et al., 2025).

Qualitative expert assessment revealed a distinct strategic shift in transgenic mice. Instead of an energy-intensive manipulative strategy (dismantling the pot into fine crumbs), 5xFAD females frequently transitioned to a passive strategy, utilizing the intact pot as a pre-formed shelter (Supplementary Figure 3). Furthermore, instances of material substitution were observed: mice unable to shred the pot incorporated standard bedding (sawdust) into the napkin matrix. This reflects impaired material discrimination and a simplification of the behavioral repertoire toward rudimentary, single-step actions.

Working memory deficits linked to hippocampal and prefrontal cortex pathology likely disrupt transitions between sub-routines, causing the animal to “forget” the overarching task goal (e.g., fragment transport) and leading to behavioral chain fragmentation. Crucially, the complete preservation of feeding behavior confirms that the decline in DBT performance reflects a loss of executive drive or apathy -as proposed by Keszycki et al. (2023) - rather than sensorimotor or masticatory dysfunction.

### Energy budget and cognitive flexibility

The hypothesis that decreased manipulative performance in the DBT is associated with general apathy or a lack of physical strength was not confirmed by the actigraphic data. Furthermore, high locomotor activity on Day 0 proved to be a negative predictor of nest quality.

If the key defect in 5xFAD females is an inability to inhibit unproductive locomotion and switch to a complex manipulative sequence, then the DBT effectively functions as a test of cognitive flexibility within a natural paradigm. Unlike traditional flexibility tests, which require food deprivation or extensive training, the DBT provides a brief, low-cost protocol with high ecological validity and minimal stress levels for the animals.

### The phenomenon of “cognitive capture” in neurodegeneration

A critical observation in our study is a population-level shift toward a hypertrophied proactive coping style in transgenic 5xFAD females. However, this “proactive” behavior likely reflects pathological disinhibition rather than an adaptive coping mechanism. The hyperlocomotion recorded during adaptation (Figure 7), combined with the dramatic decline in manipulative performance (Figures 2, 4), points to profound behavioral rigidity.

We hypothesize that the accumulation of amyloid pathology impairs prefrontal-hippocampal circuitry, compromising the animal’s capacity to inhibit a dominant response (exploratory locomotion) in favor of a more complex, cognitively demanding task (shelter construction). In this context, the DBT functions as a test of behavioral entrapment and cognitive flexibility.

The ability of a mouse to switch from initial exploratory arousal to focused, productive object manipulation represents a complex executive operation. In transgenic animals, this switching mechanism is disrupted: they remain “locked” in an unproductive locomotion cycle, serving as an early ethological marker of neurodegeneration that precedes overt memory impairment.

### Cognitive architecture of the deficit in the DBT

Nest-building behavior is sensitive to strain differences, aging, stress, and neurological disorders, serving as a reliable indicator of cognitive, affective, and sensorimotor status (Tagawa et al., 2025). Within the DBT framework, we evaluate a complex goal-directed behavior that depends critically on the functional integrity of the hippocampal-prefrontal network. Although the present study does not include direct histological validation for these cohorts, it is well-established that 15-week-old (4-month-old) 5xFAD mice already manifest robust amyloid pathology and significant synaptic loss within these specific cortical and hippocampal pathways (Oakley et al., 2006; Jawhar et al., 2012). Therefore, the behavioral deficit identified here represents an ethologically sensitive readout of early executive fragmentation, reflecting cumulative impairment across key cognitive domains:

Planning: The formation and execution of a goal-oriented sequence of actions (Miller and Cohen, 2001; Kesner and Churchwell, 2011).

Working memory: The capacity to hold and update information necessary for multi-stage manipulations (Miyake and Friedman, 2012).

Sustained attention: The capacity for prolonged concentration during object manipulation (Langner and Eickhoff, 2013).

Cognitive flexibility (task switching): The ability to interrupt unproductive behavioral patterns and return to a postponed target task (Miyake and Friedman, 2012).

In a clinical context, these executive deficits mirror the progressive decline in Activities of Daily Living (ADLs) in Alzheimer’s disease, where the disruption of complex action sequences often precedes the loss of basic motor skills.

### Limitations of the study and considerations for future research

While the DBT demonstrated high diagnostic sensitivity, several limitations warrant consideration. First, sample size reduction at 28 and 42 weeks due to longitudinal attrition limited statistical power to detect subtle genotype differences in males or main age effects within the GEE framework. Second, the pronounced female-specific phenotype restricts immediate generalization to males without dedicated follow-up cohorts.

Methodologically, spontaneous locomotor activity (SLA) was systematically recorded during the habituation phase (Day 0) to establish clean baseline profiles, but continuous actigraphy was not maintained during the manipulative phase (Days 1–3). Consequently, secondary object-related dynamics - such as neophobia or stimulus-induced hyperlocomotion - cannot be fully disentangled from cognitive-motor decline. Incorporating continuous tracking in the direct presence of the substrate will be essential in future setup iterations.

Finally, expanding the comparative ethological scope of the DBT to other rodent species (such as arboreal *Rattus rattus*) or physiological conditions characterized by heightened nesting drive (e.g., late gestation) could further refine the biological sensitivity of the assay and broaden its translational applicability across diverse neurodegenerative disease models.

## Data Availability

The raw data supporting the conclusions of this article will be made available by the authors, without undue reservation.

## Supplementary material

Supplementary Figure 1 Examples of mouse interaction with the pot at the beginning of the DBT test. **(A)** The mouse shows no interest in the pot. **(B)** The mouse is interested in interacting with the pot and actively explores it.

Supplementary Figure 2 Examples of interaction at the end of the experiment. **(A)** The animal did not gnaw the pot at all and did not build a nest. **(B)** The animal gnawed the pot only slightly and built a nest. **(C)** The animal continuously interacted with the pot and the cloth, using these items for nest construction.

Supplementary Figure 3 Other examples of behavior in the DBT. The animal sits motionless entirely within the intact pot.

Supplementary Figure 4 Examples of nests built by mice.

Supplementary Figure 5 Variations in mice’s pot-gnawing strategies.

Supplementary Figure 6 The figure shows graphs illustrating the age-related dynamics of four parameters measured in the DBT test for transgenic female mice of the 5xFAD line. **(A)** Depicts the mass loss dynamics from the initial peat pot mass over the 3-days experiment (calculated as the difference between the initial pot mass and the pot mass on day 3). **(B)** Shows the dynamics of the destroyed peat pot area over 3 days. **(C)** Presents the dynamics of nest masses obtained from the DBT test. **(D)** Illustrates the dynamics of the total area of peat pot fragments incorporated into the mice’s nests. All panels display data from the same mice at different ages. Outliers within each group are indicated by gray dots.

Supplementary Figure 7 This figure shows graphs reflecting the age-related dynamics of four DBT test parameters in wild-type (control) females of the C57BL/6J mouse strain. **(A)** Depicts the mass loss dynamics from the initial peat pot mass over the 3-days experiment (calculated as the difference between the initial pot mass and the pot mass on day 3). **(B)** Shows the dynamics of the destroyed peat pot area over 3 days. **(C)** Presents the dynamics of nest masses obtained from the DBT test. **(D)** Illustrates the dynamics of the total area of peat pot fragments incorporated into the mice’s nests. All panels display data from the same mice at different ages. Outliers within each group are indicated by gray dots.

Supplementary Figure 8 This figure shows graphs illustrating the age-related dynamics of four parameters measured in the DBT test for transgenic male mice of the 5xFAD line. **(A)** Depicts the mass loss dynamics from the initial peat pot mass over the 3-days experiment (calculated as the difference between the initial pot mass and the pot mass on day 3). **(B)** Shows the dynamics of the destroyed peat pot area over 3 days. **(C)** Presents the dynamics of nest masses obtained from the DBT test. **(D)** Illustrates the dynamics of the total area of peat pot fragments incorporated into the mice’s nests. All panels display data from the same mice at different ages. Outliers within each group are indicated by gray dots.

Supplementary Figure 9 This figure shows graphs illustrating the age-related dynamics of four parameters measured in the DBT test for wild-type (control) male mice of the C57BL/6J strain. **(A)** Depicts the mass loss dynamics from the initial peat pot mass over the 3-days experiment (calculated as the difference between the initial pot mass and the pot mass on day 3). **(B)** Shows the dynamics of the destroyed peat pot area over 3 days. **(C)** Presents the dynamics of nest masses obtained from the DBT test. **(D)** Illustrates the dynamics of the total area of peat pot fragments incorporated into the mice’s nests. All panels display data from the same mice at different ages. Outliers within each group are indicated by gray dots.
