# Supplementary material for: Impaired behavioral flexibility and coping strategy shifts in 5xFAD mice in the novel Destruction-Building Test
Source: Front Behav Neurosci. 2026 Aug 26;20:1877086. doi: 10.3389/fnbeh.2026.1877086 (PMC13553921; doi:10.3389/fnbeh.2026.1877086)
Supplement: Supplementary file 1 [file Data_Sheet_1.docx]

**Supplementary materials**

**2. Programming code**

R-code for SLA - DBT

```
> ########################################################
> # Model determinants of gnawing behavior ################
> ########################################################
> # 2. Fit GEE model with numeric cluster ID
> library(geepack)
>
> # 1. Convert subject ID to numeric format
> long_table <- long_table %>%
+   mutate(id_num = as.numeric(factor(id)))  # делает 1,2,3... для уникальных id
>
> # 2. Fit full model with all interactions
> m_gee <- geeglm(
+   WeightPPSumm ~ gene * Gender * walkS2Sway * TimeNum,
+   id = id_num,               # уже числовой
+   family = gaussian(),
+   data = long_table,
+   corstr = "exchangeable" # independence exchangeable
+ )
> # Estimate coefficients and Wald statistics
> summary(m_gee) # Individual predictor effects, including interactions

Call:
geeglm(formula = WeightPPSumm ~ gene * Gender * walkS2Sway *
    TimeNum, family = gaussian(), data = long_table, id = id_num,
    corstr = "exchangeable")

 Coefficients:
                                    Estimate   Std.err  Wald Pr(>|W|)
(Intercept)                         6.99e+00  2.13e+00 10.74   0.0010 **
geneHem                            -3.75e+00  2.21e+00  2.87   0.0901 .
Genderm                            -8.68e+00  3.13e+00  7.69   0.0056 **
walkS2Sway                         -4.12e-03  2.14e-03  3.72   0.0537 .
TimeNum                            -5.42e-02  6.75e-02  0.64   0.4221
geneHem:Genderm                     9.57e+00  3.29e+00  8.48   0.0036 **
geneHem:walkS2Sway                  2.79e-03  2.20e-03  1.60   0.2060
Genderm:walkS2Sway                  8.66e-03  3.87e-03  5.01   0.0252 *
geneHem:TimeNum                    -2.26e-02  7.00e-02  0.10   0.7462
Genderm:TimeNum                     1.92e-01  1.02e-01  3.56   0.0593 .
walkS2Sway:TimeNum                  4.91e-05  7.56e-05  0.42   0.5158
geneHem:Genderm:walkS2Sway         -1.05e-02  4.02e-03  6.76   0.0093 **
geneHem:Genderm:TimeNum            -1.98e-01  1.07e-01  3.43   0.0640 .
geneHem:walkS2Sway:TimeNum         -1.60e-05  7.70e-05  0.04   0.8351
Genderm:walkS2Sway:TimeNum         -2.47e-04  1.35e-04  3.38   0.0659 .
geneHem:Genderm:walkS2Sway:TimeNum  2.92e-04  1.39e-04  4.42   0.0356 *
---
Signif. codes:  0 ‘***’ 0.001 ‘**’ 0.01 ‘*’ 0.05 ‘.’ 0.1 ‘ ’ 1

Correlation structure = exchangeable
Estimated Scale Parameters:

            Estimate Std.err
(Intercept)    0.661   0.104
  Link = identity

Estimated Correlation Parameters:
      Estimate Std.err
alpha        0       0
Number of clusters:   123  Maximum cluster size: 1
> anova(m_gee, Wald = FALSE) # Cluster-wise significance: main effects + associated interactions
Analysis of 'Wald statistic' Table
Model: gaussian, link: identity
Response: WeightPPSumm
Terms added sequentially (first to last)

                               Df   X2 P(>|Chi|)
gene                            1 39.2   3.8e-10 ***
Gender                          1 10.6    0.0011 **
walkS2Sway                      1 22.5   2.1e-06 ***
TimeNum                         1  0.7    0.4008
gene:Gender                     1 27.0   2.0e-07 ***
gene:walkS2Sway                 1  4.7    0.0302 *
Gender:walkS2Sway               1  0.2    0.6215
gene:TimeNum                    1  3.2    0.0755 .
Gender:TimeNum                  1  0.3    0.5667
walkS2Sway:TimeNum              1  2.4    0.1205
gene:Gender:walkS2Sway          1  2.5    0.1105
gene:Gender:TimeNum             1  0.0    0.9280
gene:walkS2Sway:TimeNum         1  1.6    0.2094
Gender:walkS2Sway:TimeNum       1  0.1    0.7042
gene:Gender:walkS2Sway:TimeNum  1  4.4    0.0356 *
---
Signif. codes:  0 ‘***’ 0.001 ‘**’ 0.01 ‘*’ 0.05 ‘.’ 0.1 ‘ ’ 1
>
> # 3. Check for multicollinearity
> library(car)
> vif(m_gee)
there are higher-order terms (interactions) in this model
consider setting type = 'predictor'; see ?vif
gene (339)  Gender (959)  walkS2Sway (481) TimeNum (201)  gene:Gender (805)  gene:walkS2Sway (894)  Gender:walkS2Sway (949) gene:TimeNum  (474) Gender:TimeNum  (961) walkS2Sway:TimeNum  (1470)  gene:Gender:walkS2Sway (853) gene:Gender:TimeNum (831) gene:walkS2Sway:TimeNum (1947)     Gender:walkS2Sway:TimeNum (1144) gene:Gender:walkS2Sway:TimeNum (1062)
> # High VIF values detected for multiple terms; simplify model structure...
>
> # 4. Fit reduced model
> m_gee <- geeglm(
+   WeightPPSumm ~ gene + Gender + walkS2Sway + TimeNum+ gene:Gender,
+   id = id_num,               # уже числовой
+   family = gaussian(),
+   data = long_table,
+   corstr = "exchangeable" # independence exchangeable
+ )
>
> # 5. Re-evaluate multicollinearity
> library(car)
> vif(m_gee)
there are higher-order terms (interactions) in this model
consider setting type = 'predictor'; see ?vif
       gene      Gender  walkS2Sway     TimeNum gene:Gender
       2.66        4.93        1.55        1.12        4.75
> # VIF within acceptable range

> # Estimate coefficients
> summary(m_gee) # Individual predictor effects

Call:
geeglm(formula = WeightPPSumm ~ gene + Gender + walkS2Sway +
    TimeNum + gene:Gender, family = gaussian(), data = long_table,
    id = id_num, corstr = "exchangeable")

 Coefficients:
                 Estimate   Std.err   Wald Pr(>|W|)
(Intercept)      4.265877  0.407386 109.65  < 2e-16 ***
geneHem         -1.884612  0.239510  61.91  3.6e-15 ***
Genderm         -1.735841  0.287593  36.43  1.6e-09 ***
walkS2Sway      -0.001198  0.000302  15.69  7.5e-05 ***
TimeNum         -0.006931  0.007000   0.98     0.32
geneHem:Genderm  1.613572  0.310406  27.02  2.0e-07 ***
---
Signif. codes:  0 ‘***’ 0.001 ‘**’ 0.01 ‘*’ 0.05 ‘.’ 0.1 ‘ ’ 1

Correlation structure = exchangeable
Estimated Scale Parameters:

            Estimate Std.err
(Intercept)    0.762   0.116
  Link = identity

Estimated Correlation Parameters:
      Estimate Std.err
alpha        0       0
Number of clusters:   123  Maximum cluster size: 1
> anova(m_gee, Wald = FALSE) # Cluster-wise significance
Analysis of 'Wald statistic' Table
Model: gaussian, link: identity
Response: WeightPPSumm
Terms added sequentially (first to last)

            Df   X2 P(>|Chi|)
gene         1 39.2   3.8e-10 ***
Gender       1 10.6    0.0011 **
walkS2Sway   1 22.5   2.1e-06 ***
TimeNum      1  0.7    0.4008
gene:Gender  1 27.0   2.0e-07 ***
---
Signif. codes:  0 ‘***’ 0.001 ‘**’ 0.01 ‘*’ 0.05 ‘.’ 0.1 ‘ ’ 1

> # Proportion of explained variance
> m_lm <- lm(WeightPPSumm ~ gene + Gender + walkS2Sway + TimeNum + gene:Gender, data = long_table)
> summary(m_lm)$r.squared
[1] 0.505
>

> # Baseline contribution ("mice gnaw regardless of predictors")
> anova_type3 <- Anova(m_lm, type = 3)
> ss_all      <- anova_type3$`Sum Sq`
> ss_intercept <- ss_all[rownames(anova_type3) == "(Intercept)"]
> ss_total    <- sum(ss_all)
> prop_intercept <- ss_intercept / ss_total
> prop_intercept
[1] 0.332
>
> # Relative contribution of predictors to explained variance
> pred_mask  <- !rownames %in% c("(Intercept)", "Residuals")
> ss_model   <- sum(ss_all[pred_mask])
> propPart_type3 <- ss_all[pred_mask] / ss_model
> names(propPart_type3) <- rownames[pred_mask]
> propPart_type3
       gene      Gender  walkS2Sway     TimeNum gene:Gender
    0.37926     0.36518     0.09391     0.00505     0.15660
```
